# Thermal Decomposition In Situ Monitoring System of the Gas Phase Cyclopentadienyl Tris(dimethylamino) Zirconium (CpZr(NMe_2_)_3_) Based on FT-IR and QMS for Atomic Layer Deposition

**DOI:** 10.1186/s11671-020-03400-2

**Published:** 2020-09-04

**Authors:** Jong-Ki An, Eunmi Choi, Seob Shim, Hayeong Kim, Goru Kang, Ju-Young Yun

**Affiliations:** 1 New Product Development Department, Wonik IPS, 75, Jinwisandan-ro, Jinwi-myeon, Pyeongtaek-si, Gyeonggi-do 17709 Republic of Korea; 2 Vacuum Materials Measurement Team, Korea Research Institute of Standards and Science (KRISS), 267 Gajeong-ro, Yuseong-gu, Daejeon, 34113 Republic of Korea; 3 Liquid Processing & Casting R&D Group, Korea Institute of Industrial Technology, 156, Gaetbeol-ro, Yeonsu-gu, Incheon, 21999 Republic of Korea; 4 Nanomaterials Science & Engineering, University of Science and Technology (UST), Daejeon, 34113 Republic of Korea

**Keywords:** Thermal properties, In situ monitoring, FTIR&QMS, Precursor, CpZr[N(CH_3_)_2_]_3_

## Abstract

We developed a newly designed system based on in situ monitoring with Fourier transform infrared (FT-IR) spectroscopy and quadrupole mass spectrometry (QMS) for understanding decomposition mechanism and by-products of vaporized Cyclopentadienyl Tris(dimethylamino) Zirconium (CpZr(NMe_2_)_3_) during the move to process chamber at various temperatures because thermal decomposition products of unwanted precursors can affect process reliability. The FT-IR data show that the –CH_3_ peak intensity decreases while the –CH_2_– and C=N peak intensities increase as the temperature is increased from 100 to 250 °C. This result is attributed to decomposition of the dimethylamido ligands. Based on the FT-IR data, it can also be assumed that a new decomposition product is formation at 250 °C. While in situ QMS analysis demonstrates that vaporized CpZr(NMe_2_)_3_ decomposes to N-ethylmethanimine rather than methylmethyleneimine. The in situ monitoring with FT-IR spectroscopy and QMS provides useful information for understanding the behavior and decomposes of CpZr(NMe_2_)_3_ in the gas phase, which was not proven before. The study to understand the decomposition of vaporized precursor is the first attempt and can be provided as useful information for improving the reliability of a high- advanced ultra-thin film deposition process using atomic layer deposition in the future.

## Introduction

The ongoing drive to miniaturize electronic devices has led to issues regarding reliability due to the increased leakage of current by direct tunneling [1]. To solve this problem, high-k materials having wide band gaps and high dielectric constants, such as Al_2_O_3_, Y_2_O_3_, HfO_2_, and ZrO_2_, are used [2]. ZrO_2_ is especially useful as an insulating layer and dielectric owing to its wide band gap (3.4 eV at room temperature), high refractive index, suitable band offset on Si, acceptably low leakage current, and good thermal stability [3, 4]. For these reasons, this material is widely used in gas sensors and optoelectronic devices [5].

Ultra-thin ZrO_2_ films are typically deposited by chemical vapor deposition (CVD) [3, 6] or atomic layer deposition (ALD) [7, 8]. Of the two, ALD (which is based on self-limiting reactions and alternating surface control technologies) has many advantages, including control over the layer thickness on the sub-nm level via layer-by-layer growth, highly uniform thin film deposition, the formation of defect-free structures, and good reproducibilit y[9]. As such, ALD is the primary technique used in the fabrication of nanoscale devices [6].

Because thin film growth based on ALD proceeds via the chemical reactions of vaporized precursors and co-reactants, it is important to select the appropriate precursor for a successful process [10, 11]. In general, an ALD metal precursor should have a high vapor pressure, a high degree of purity and low viscosity, and superior chemical and thermal stability. The thermal stability of the precursor is particularly important, because the precursor is held at a high temperature in the bubbling container while being vaporized and also exposed to elevated temperatures in the gas line that feeds the vaporized precursor into the chamber [12].

Thermal stability of the precursor for ALD process has to be considered in two points. Precursors are exposed to consistently thermal stress because precursors are heated to be vaporized during ALD process. Also, gas line for feeding vaporized precursors into the chamber is heated above the vaporizer temperature to prevent condensation or coagulation of the vaporized precursors and facilitate diffusion. At this time, the gas line is heated to a higher temperature as it is closer to the chamber from the evaporator. It is generally heated to a temperature between 100 and 200 °C. Therefore, high thermal stability should be ensured so that thermal decomposition does not occur in this temperature range. But, the precursor that has too high thermal stability does not decompose ligand at process temperature and it leads to degradation of film reliability [13]. So, thermal decomposition of ligand should occur at process temperature. In order to two conflicting requirements about thermal stability of precursor, many researchers are designing a new structure precursor [14–16].

The thermal stability and the decomposition mechanism of precursor have been studied by thermogravimetric analyzer (TGA), differential scanning calorimeter (DSC), Fourier transform infrared (FT-IR) and spectroscopy, and quadrupole mass spectroscopy (QMS) [17–22]. TGA and DSC are used primarily to quickly identify the process window of a precursor because it provides information on the thermal properties of materials. FT-IR and QMS are used to understand the reaction of precursors. Mostly studies on decomposition mechanisms of precursor using FT-IR and QMS have been used to understand reaction mechanisms on the deposition substrates [23, 24]. FT-IR quickly provides information on the precise data of precursor or its chemical reaction [12], and QMS make know the gaseous species that relate to surface reaction [25, 26]. However, FT-IR spectra have possibility multiple overlapping because many IR-active species are generated during ALD process [27], and a variety of molecules with the same QMS measurement value present because QMS is detected by electron impact from a filament [26, 28]. The combination of FT-IR and QMS is very useful to complement their respective weakness and help understand the thermal stability and the decomposition mechanism of precursor.

Thermal decomposition in moving vaporized precursors due to thermal stress exposure has not received attention. But, understanding this mechanism can provide very useful information for process optimization and new precursor design because thermal decomposition of unwanted precursors can affect process reliability.

In this study, we try to understand about the thermal decomposition mechanism of the typical precursor Cyclopentadienyl Tris(dimethylamino) Zirconium (CpZr(NMe_2_)_3_; Cp: cyclopentadienyl (C_5_H_5_), Me: methyl (-CH_3_) in gas phase by using newly designed system based on in situ monitoring using Fourier transform infrared (FT-IR) spectroscopy and quadrupole mass spectrometry (QMS).

## Experimental Methods

### The Newly Designed In Situ Monitoring Systems

**Fig. 1:**
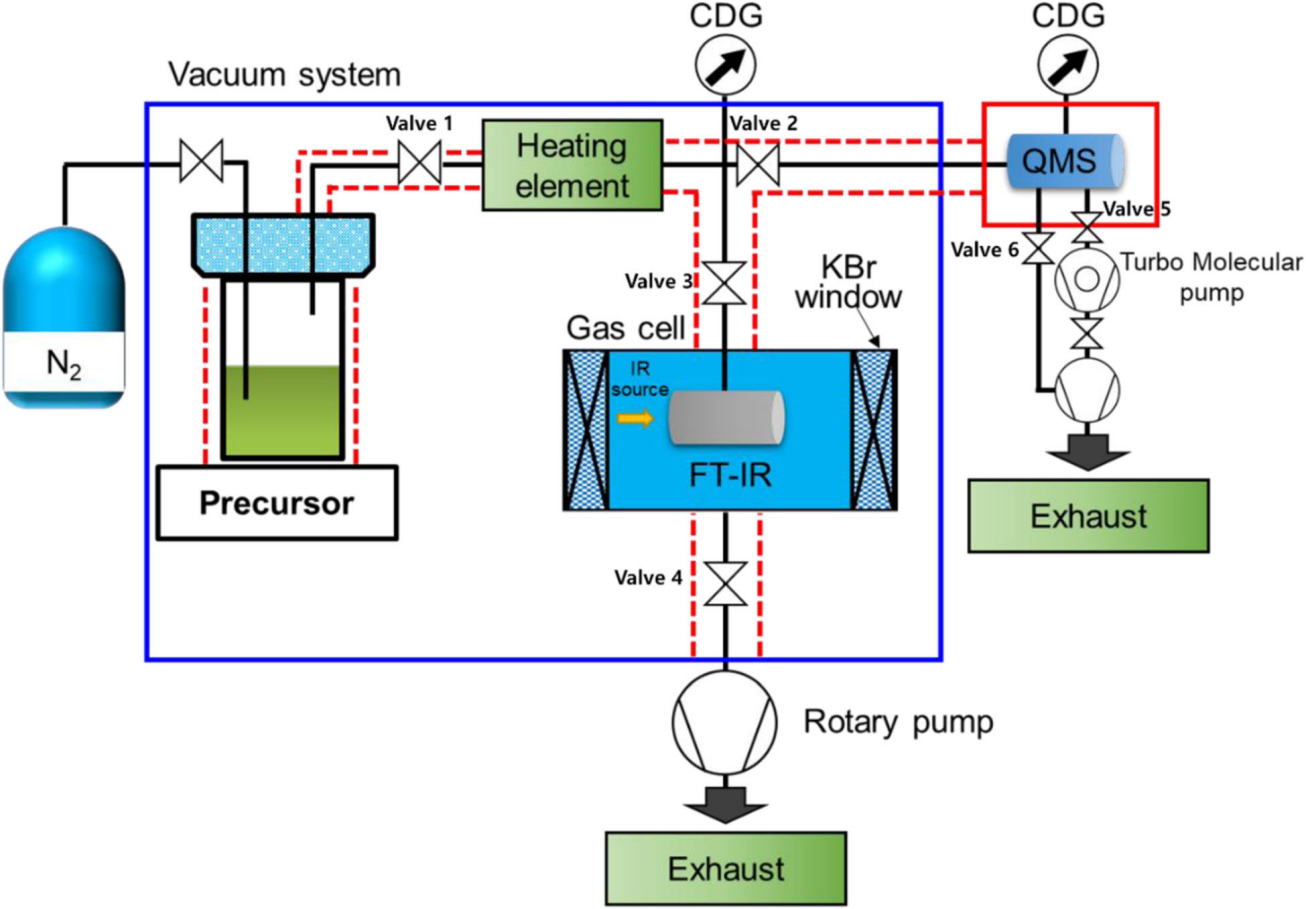
Schematic of the newly designed in-situ monitoring systems

Figure 1 shows a schematic of the in situ monitoring system designed to examine the thermal decomposition of vaporized precursors. In this study, the in situ monitoring systems are newly designed to observe the behavior of the precursor exposed to the thermal stress in the vapor.

This apparatus comprises a vacuum system connected to a bubbler, a feed line with a valve, a heating element, and a gas cell attached to an FT-IR spectrometer (Nicolet, Avatar 360). The lab-made vacuum system was held at a pressure of 10^−3^ Torr using a rotary pump and the pressure level was measured with a capacitance diaphragm gauge (CDG) having a 10-Torr range. In each trial, the precursor was placed inside the bubbler (which was held at 80 °C), after which the vaporized material was supplied to the feed line, which was constructed of stainless steel (SUS) and had stainless steel gaskets at each connection in addition to manual valves that allowed for the application of vacuum. Both the feed line and FT-IR gas cell were held at 100 °C to prevent the precursor from condensing. The gas cell was constructed of KBr windows, had a volume of 45.5 mL, and was also held at a vacuum of 10^−3^ Torr. The FT-IR employed a Hg-Cd-Te detector and cooled with liquid nitrogen. The spacing between the IR source and KBr window is 5 cm. A specially designed heating element allowed the temperature of the vaporized precursor to be controlled up to 500 °C.

A QMS instrument (Inficon, Transpector CPM) was also connected to the vacuum system to allow for additional analysis of the vaporized precursor and was maintained at 150 °C with a heating jacket. The internal pressure in the QMS instrument was kept at 10^−8^ Torr using a combination of a turbomolecular pump and a rotary pump, and this pressure was monitored with a CDG.

### Characterization Methods

The CpZr(NMe_2_)_3_ (Soulbrain, 99.8%) was vaporized by heating the stainless steel bubbler. During the initial 10 min, all valves of in situ monitoring systems were opened and the vaporized CpZr(NMe_2_)_3_ was passed to get identical vaporized precursor. Valves 2 and 3 were closed and the resulting vapors were transferred to the high-temperature zone. Valves 1, 2, and 3 were closed to isolate the vaporized CpZr(NMe_2_)_3_ for 5 min in heating element to give enough thermal stress. At that time, the heating element was heated to temperatures from 100 to 250 °C in increments of 25 °C. Valves 2 and 3 were opened, and 4, 5, and 6 were closed to move decomposition products to FT-IR and QMS. Valves 2 and 3 were closed and the resulting decomposition products were assessed using both the FT-IR and QMS instrumentation. The FT-IR acquired spectra over the range from 500 to 3500 cm^−1^ with a spectral resolution of 8 cm^−1^, summing 32 scans to obtain each spectrum. The QMS analyzed ions over the range from 1 to 300 atomic mass units (amu), operating in the electron impact ionization mode with an ionization energy of 30 eV. After measured, Valves 4, 5, and 6 were opened to remove the heated CpZr(NMe_2_)_3_ for 10 min.

## Results and Discussion

The precursor including cyclopentadienyl moieties tends to exhibit high thermal stability owing to the strong bonding between these groups and metal atoms, and CpZr(NMe_2_)_3_ is typically processed at temperatures from 300 to 380 °C [29–31].

Therefore, the thermal decomposition of vaporized CpZr(NMe_2_)_3_ was expected to proceed primarily at the trimethylamine ligands rather than at the cyclopentadienyl-Zr bonds. Based on this assumption, the expected main decomposition products are summarized in Table 1 [32–34]. And, the mechanisms of expected main decomposition products are as follows: 
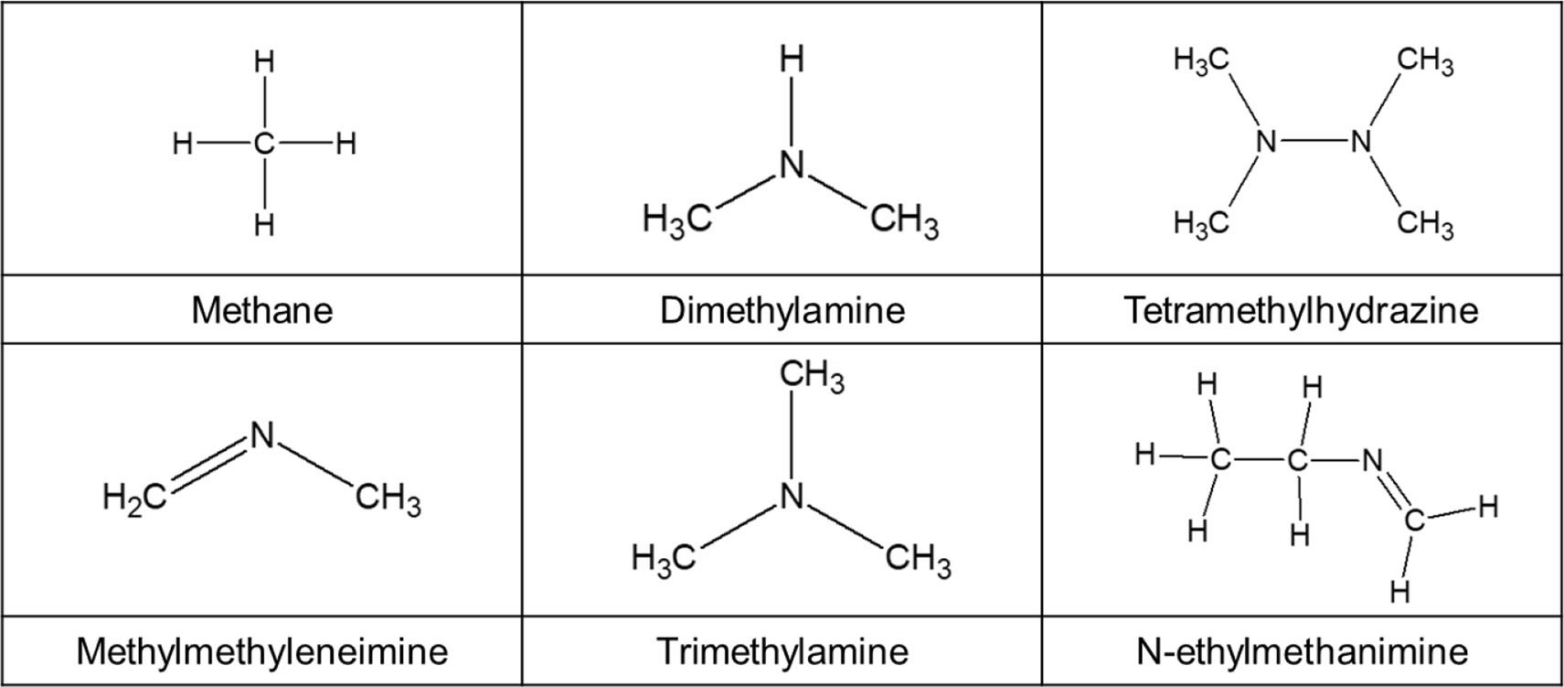

**Table 1:**
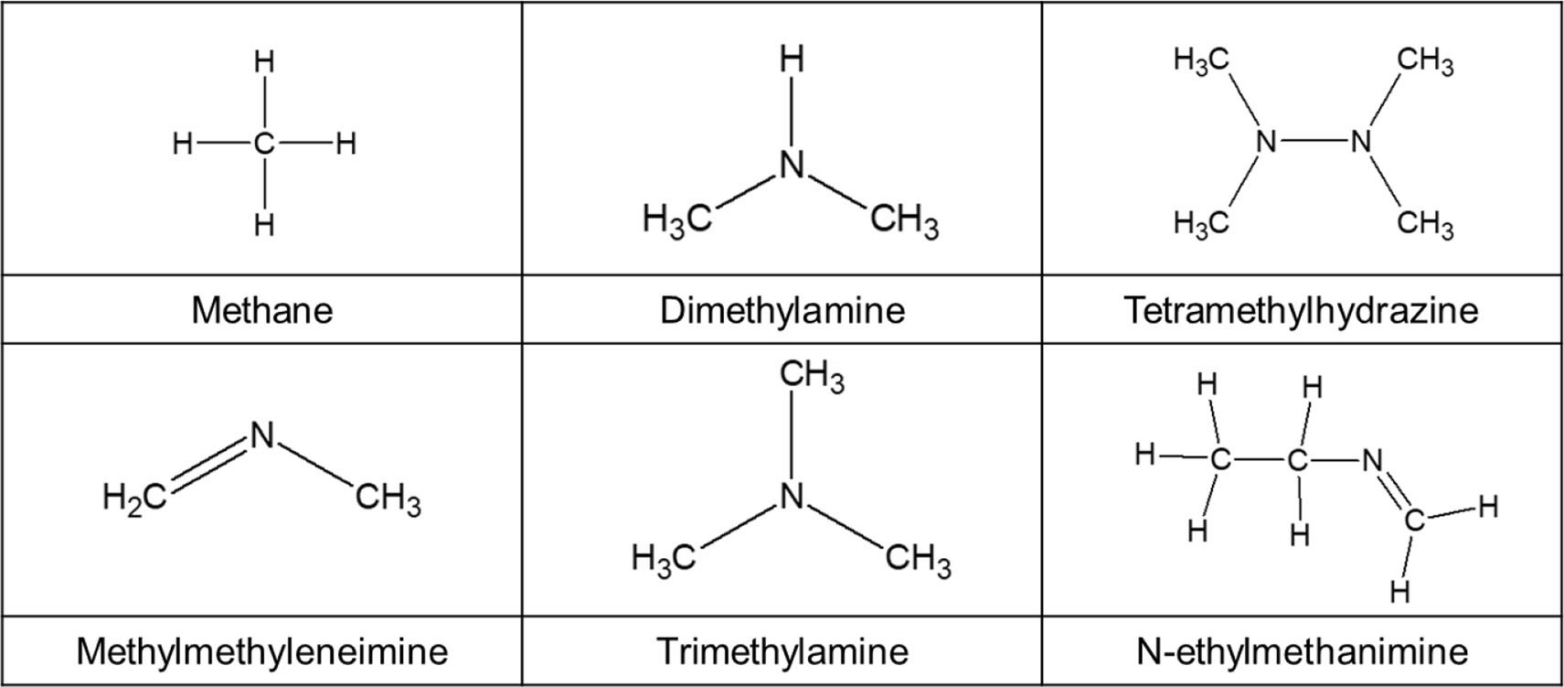
The expected thermal decomposition product of CpZr(NMe_2_)_3_

Hydrogenation reaction of dimethylamino ligand

H_2_ + •NMe_2_ → H• + HNMe_2_

Hydrogen Elimination reaction of dimethylamino ligand

•NMe_2_ + •NMe_2_ → HNMe_2_ + CH_2_ = N-CH_3_

Covalent bonding of dimethylamino ligand

•NMe_2_ + •NMe_2_ → (CH_3_)_2_N-N(CH_3_)_2_

1,2-elimination reaction of Tetramethylhydrazine

(CH_3_)_2_N-M(NCH_2_)_3_ → N(CH_3_)_3_ + H_3_C-N = M(NMe_2_)_2_

**Fig. 2:**
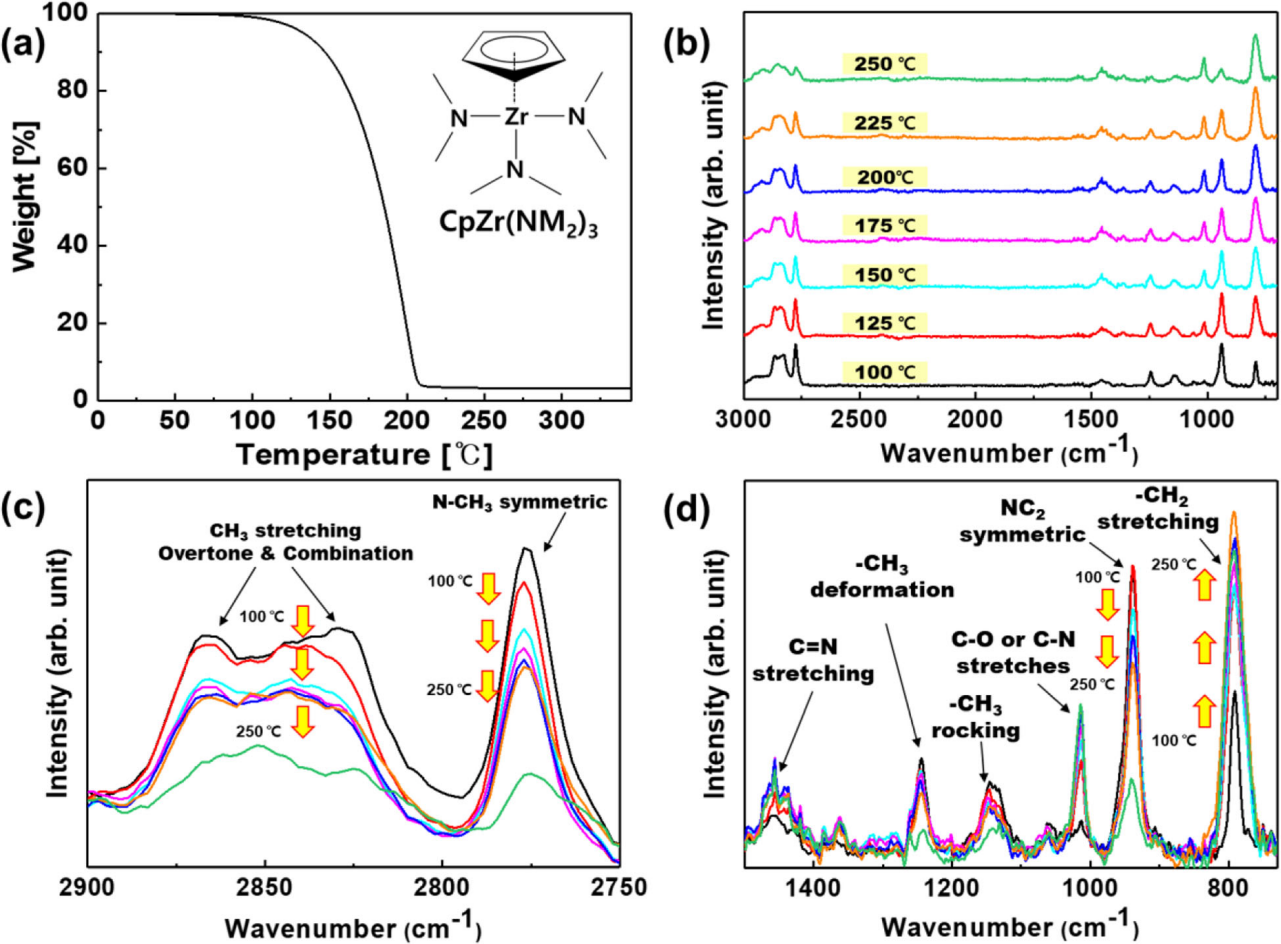
TGA result with chemical structure and FT-IR spectrum from 100 to 250 °C of CpZr(NMe_2_)_3_. **a** Chemical structure, FT-IR spectrum, **b** full range of 3000~700 cm^−1^, **c** range of 3000~2750 cm^−1^, and **d** range of 1300~700 cm^−1^

Figure 2 a shows the TGA result with molecular structure of CpZr(NMe_2_)_3_, while Fig. 2 b–d presents the FT-IR spectra acquired at various decomposition temperatures.

The TGA analysis result shows the approximate thermal decomposition temperature of CpZr(NMe_2_)_3_. The occurred temperatures in which the mass loss are0.5% and 5% are 87.6 °C and 132.6 °C, respectively. We also confirm that the weight became not zero at high temperature. It means that the Cp Zr(NMe_2_)_3_ is not completely decomposed and non-volatile materials are generated and remain.

**Fig. 3:**
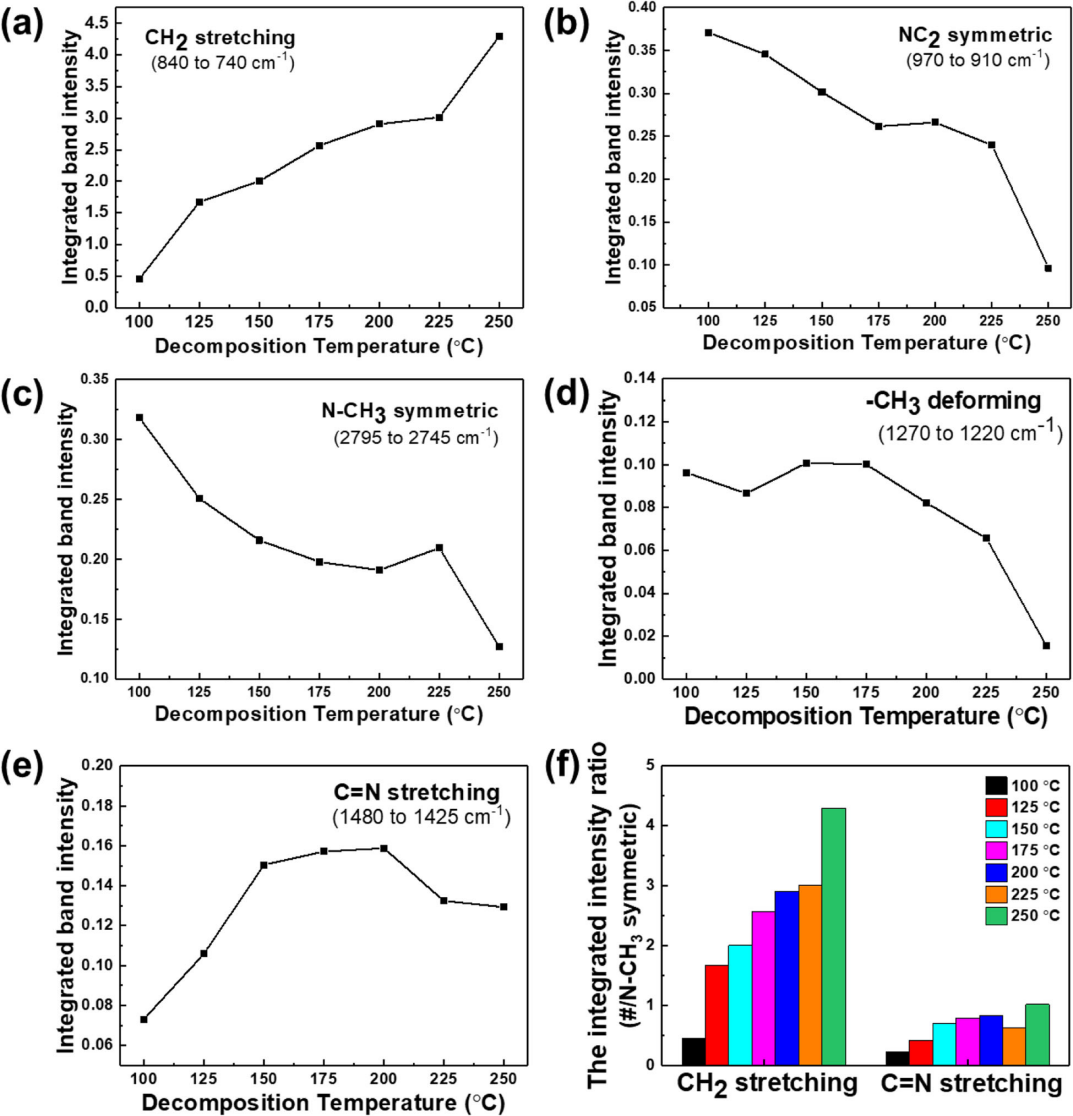
Integrated band intensity and normalized of CpZr(NMe_2_)_3_ spectra main peak focus on spectra region **a** –CH_2_– stretching (840 to 740 cm^−1^), **b** NC_2_ symmetric (970 to 910 cm^−1^), **c** N–CH_3_ symmetric (2795 to 2745 cm^−1^), **d** –CH_3_ deforming (1270 to 1220 cm^−1^) and **e** C=N stretching (1480 to 1425 cm^−1^) bands, **f** the integrated intensity ratio of main peak/N–CH_3_ peak areas

The primary absorption peaks generated by the gas phase CpZr(NMe_2_)_3_ were associated with alkane C–H stretching (around 3000–2840 cm^−1^), N–CH_3_ symmetric stretching (2776 cm^−1^), C=N stretching (around 1500–1400 cm^−1^), –CH_3_ deformation (1241 cm^−1^), –CH_3_ rocking (1142 cm^−1^), NC_2_ symmetric stretching (939 cm^−1^), and –CH_2_– stretching (794 cm^−1^). As the temperature of the vapor was increased from 100 to 250 °C, the intensities of these main absorption peaks all decreased, except for the peak related to –CH_2_– stretching (Fig. 3). As noted above, in the case of CpZr(NMe_2_)_3_, the trimethylamine ligand is more readily thermally decomposed than the cyclopentadienyl ligand, due to the difference in the dissociation energies of the Zr–N and C–N bonds. Therefore, it was expected that dimethylamine would be produced by the hydrogenation reaction of the dimethylamido ligand as a result of cleavage of the Zr–N bonds in the compound. It is also likely that C=N bonds were formed by the hydrogen elimination reaction of the dimethylamido ligands, because the intensity of the FT-IR peak associated with C=N bond stretching at 1450 cm^−1^ was increased with increasing temperature (Fig. 3 e), in agreement with the predicted product in Table 1.

The intensity of the peak resulting from –CH_3_ deformation was sharply decreased at 250 °C, and the intensities of the other peaks associated with –CH_3_ groups also changed abruptly (Fig. 3 c, d). In addition, when the –CH_2_– stretching and C=N stretching peak intensities are normalized to the N–CH_3_ peak intensity, the –CH_2_– peak is seen to abruptly increase in intensity at 250 °C. These data demonstrate that the decomposition of –CH_3_ groups proceeds rapidly at 250 °C and that the reaction that produces –CH_2_– groups proceeds more readily than at other temperatures. In contrast, the C=N stretching peak intensity is not significantly increased compared with the increase in the –CH_2_– peak (Fig. 3 f). Therefore, it appears that the decomposition reactions of CpZr(NMe_2_)_3_ at 250 °C differ from those in the previously proposed mechanism, in which the decomposition reactions of the dimethylamido ligands, such as hydrogenation and hydrogen elimination, predominate (Fig. 3 f).

**Fig. 4:**
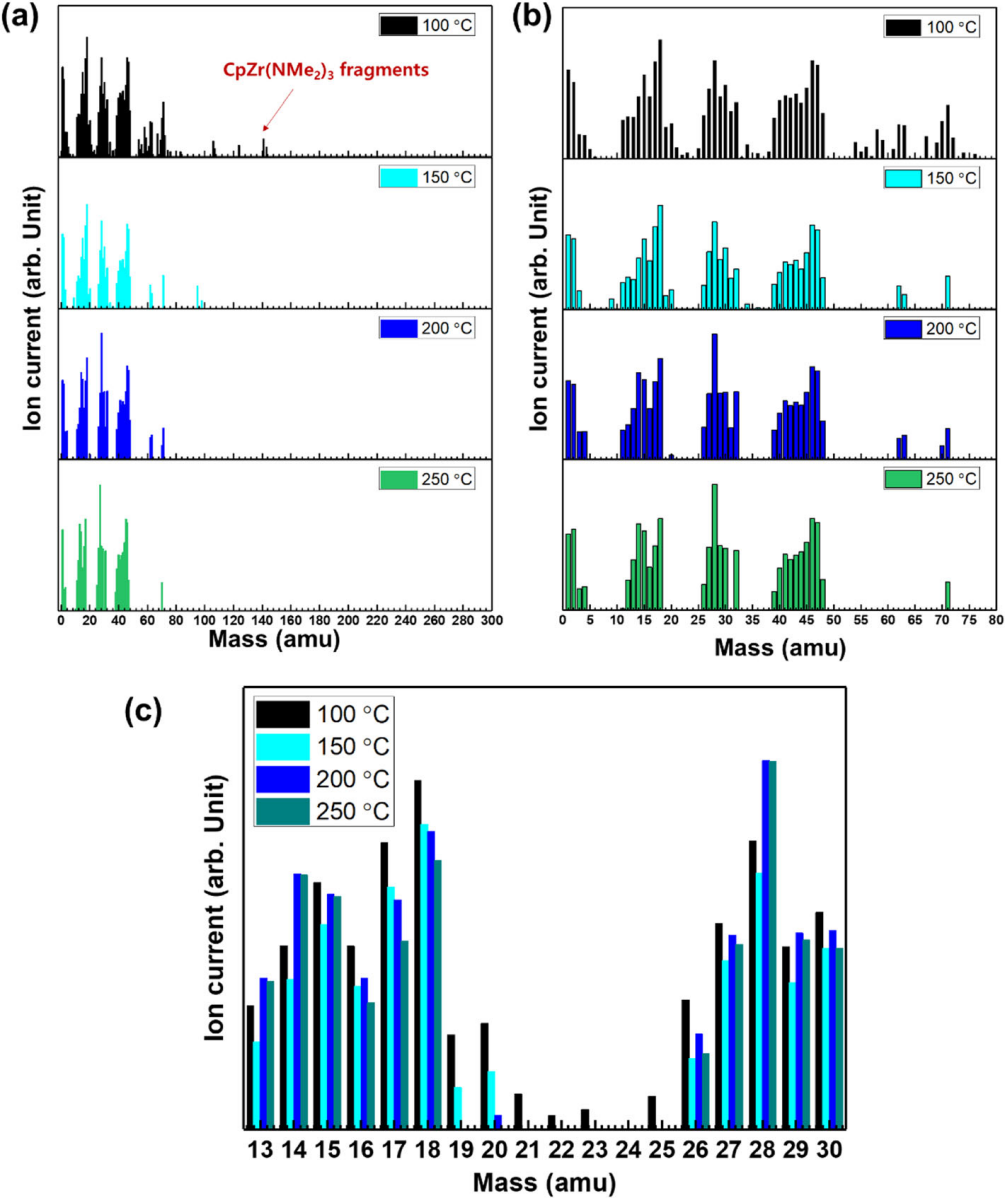
QMS analysis data of CpZr(NMe_2_)_3_ **a** 0 to 300 amu, **b** 0 to 80 amu, and **c** 13 to 30 amu

Figure 4 provides the QMS analysis data. The QMS used in this study was able to monitor ions up to 300 amu, and CpZr(NMe_2_)_3_ and its fragments have masses of 228 and 144 amu, respectively. However, the CpZr(NMe_2_)_3_ parent ion at 144 amu was only detected at a heating temperature of 100 °C. Above this temperature, fragments with masses less than 80 amu were observed as a result of the rapid decomposition of the vaporized CpZr(NMe_2_)_3_ (Fig. 4 a).

Table 2 summarizes the thermal decomposition products obtained from the CpZr(NMe_2_)_3_. The ions appearing at 5 amu and below are attributed to hydrogen and hydrogen ionization, from 10–20 amu to methane and methane ionization, from 25–32 amu to N-ethylmethanimine, trimethylamine fragments and ionization, from 40–50 amu to dimethylamine and N-ethylmethanimine ionization, and from 60–75 amu to cyclopentadienyl and cyclopentadienyl ionization (Fig. 4 b).

**Table 2:** Mass per amu of CpZr(NMe_2_)_3_ main thermal decomposition product and its fragment

Mass/amu	Decomposition product
1	Hydron
2	Hydrogen
15	Methyl
16	Methane
22	Methylmethyleneimine fragment
28	N-ethylmethanimine fragment
30	Trimethylamine fragment
43	Methylmethyleneimine
44	Tetramethylhydrazine fragment
45	Dimethylamine
57	N-ethylmethanimine
59	Trimethylamine
65	Cyclopentadienyl
88	Tetramethylhydrazine

The FT-IR spectra suggest that –CH_3_ groups decompose to –CH_2_– and that C=N bonds are formed, while other decomposition reactions also proceed at or above 250 °C. Figure 4 c shows that the masses attributed to –CH_2_– (13, 14, and 15 amu) increase with increasing heating temperature, while the masses associated with methane and methyl ionization (16, 17, and 18 amu) decrease with increasing heating temperature. Therefore, these results confirm that both the FT-IR and QMS analyses provide similar results regarding the decomposition reactions of vaporized CpZr(NMe_2_)_3_.

**Fig. 5:**
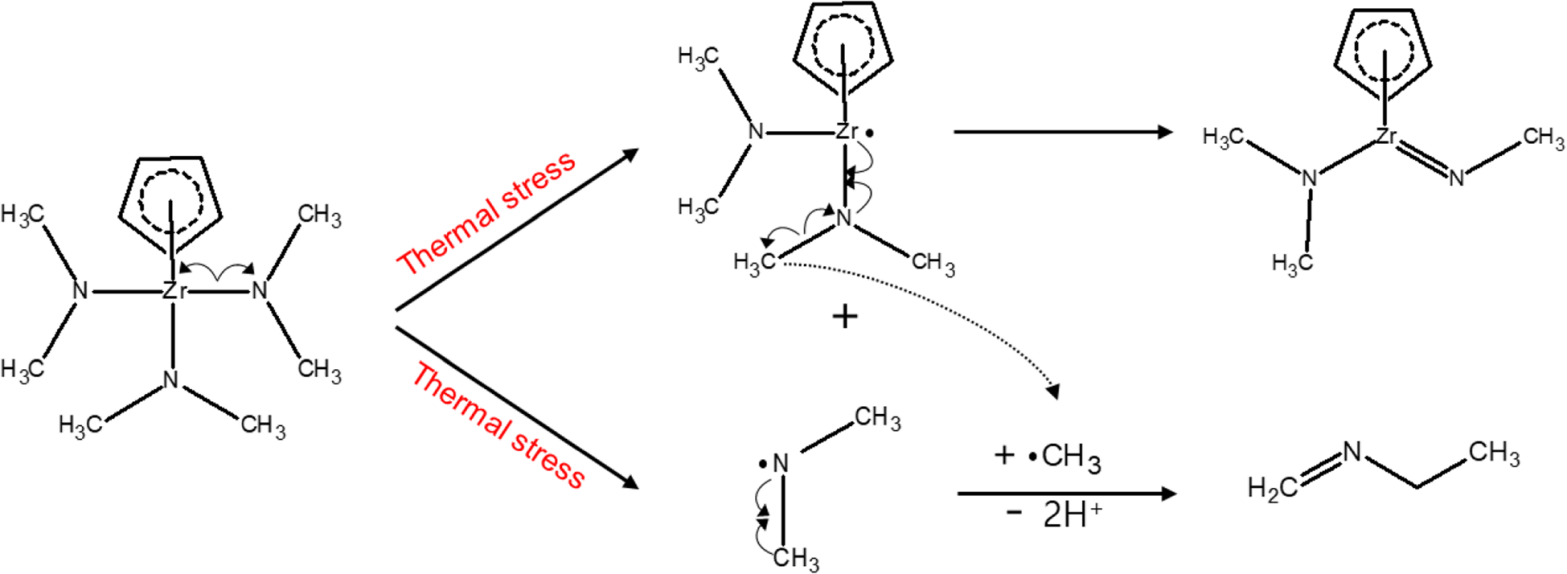
The expected main decomposition mechanisms of CpZr(NMe_2_)_3_ on gas phases

The expected decomposition products that have C=N bonds are methylmethyleneimine and N-ethylmethanimine, which would appear at mass values of 22 and 28 amu, respectively. The results of the QMS analysis show that the 21, 22, and 23 amu peaks were either weak or absent, while the 27, 28, and 29 amu peaks were strong and increased in intensity with increasing temperature (Fig. 4 c). These results confirm the decomposition mechanism suggested by the FT-IR analysis, in which the dimethylamido ligand is decomposed to generate different decomposition products depending on heating temperature. Based on the analysis data, we forecast the main decomposition mechanism of CpZr(NMe_2_)_3_ on gas phase (Fig. 5).

When CpZr(NMe_2_)_3_ is exposed to heating temperatures under 250 °C, it has different two decomposition mechanisms. Methylmethyleneimine that may fail to react further due to insufficient thermal energy and N-ethylmethanimine are mixed at temperatures 100 °C and below, and N-ethylmethanimine is present only at temperatures above 100 °C. When CpZr(NMe_2_)_3_ is exposed to heating temperatures 250 °C and above, N-ethylmethanimine and unknown decomposition products containing CH_2_ bonding are produced as decomposition products.

It can confirm from the analysis that when CpZr(NMe_2_)_3_ is exposed to thermal stress, methylmethyleneimine is preferentially generated, N-ethylmethanimine is generated through additional reactions, and unknown decomposition products containing CH_2_ bonding are generated when exposed to higher thermal energy above 250 °C. Therefore, we can easily predict methylmethyleneimine at temperatures lower than 100 °C and unknown decomposition products containing CH_2_ bonding at temperatures above 250 °C exist as a major decomposition.

The thermal decomposition mechanism of vaporized CpZr(NMe_2_)_3_, which has been confirmed by using our newly developed in situ monitoring systems, is expected to provide very useful information for ALD process optimization and new precursor design. Also, we try to suggest that expected mechanisms, and it showed that the in situ monitoring systems are useful to understand the reaction mechanism including thermal decomposition in the gas phase of various materials.

## Conclusion

This work developed an in situ monitoring system using FT-IR and QMS and applied this new technique to assess the decomposition of vaporized CpZr(NMe_2_)_3_. FT-IR analysis determined that the trimethylamine ligands were decomposed and that dimethylamine was formed via a hydrogenation reaction. The data also demonstrate that –CH_2_– and C=N groups were generated by elimination reactions as the temperature was increased. However, the in situ FT-IR analysis was unable to confirm which product having C=N bonds were obtained from the decomposition of dimethylamido ligands. QMS data demonstrated that N-ethylmethanimine was produced to a much greater extent than methylmethyleneimine via decomposition of the dimethylamido ligands as the temperature was increased.

As the result, we estimated the reaction mechanism by analyzing the decomposition products of vaporized CpZr(NMe_2_)_3_ by thermal stress using FT-IR and confirmed that the decomposition product with C=N groups is N-ethylmethanimine by using QMS analysis. The thermal decomposition mechanism of vaporized CpZr(NMe_2_)_3_ provides very useful information for optimization of ALD process and new precursor design.

## Data Availability

All data supporting the conclusions of this article are included within the article.

## Abbreviations

CpZr(NMe_2_)_3_: Cyclopentadienyl Tris(dimethylamino) Zirconium
FT-IR: Fourier transform infrared spectroscopy
QMS: Quadrupole mass spectrometry
CVD: Chemical vapor deposition
ALD: Atomic layer deposition
TGA: Thermogravimetric analyzer
DSC: Differential scanning calorimeter
